# Intra-abdominal infections: the role of different classifications on the selection of the best antibiotic treatment

**DOI:** 10.1186/s12879-019-4604-0

**Published:** 2019-11-21

**Authors:** João Silva-Nunes, Teresa Cardoso

**Affiliations:** 1 Instituto de Ciências Biomédicas Abel Salazar, Universidade do Porto (ICBAS-UP), Rua de Jorge Viterbo Ferreira n.º 228, Porto, 4050-313 Portugal; 2 Intensive Care Unit, Unidade de Cuidados Intensivos Polivalente, Hospital de Santo António - Centro Hospitalar Universitário do Porto (HSA-CHUP), Largo Prof. Abel Salazar, Porto, 4099-001 Portugal

**Keywords:** Intra-abdominal infections, Classification, Antibiotic therapy, Hospital mortality

## Abstract

**Background:**

Intra-abdominal infections (IAIs) represent a most frequent gastrointestinal emergency and serious cause of morbimortality. A full classification, including all facets of IAIs, does not exist. Two classifications are used to subdivide IAIs: uncomplicated or complicated, considering infection extent; and community-acquired, healthcare-associated or hospital-acquired, regarding the place of acquisition. Adequacy of initial empirical antibiotic therapy prescribed is an essential need. Inadequate antibiotic therapy is associated with treatment failure and increased mortality. This study was designed to determine accuracy of different classifications of IAIs to identify infections by pathogens sensitive to current treatment guidelines helping the selection of the best antibiotic therapy.

**Methods:**

A retrospective cohort study including all adult patients discharged from hospital with a diagnosis of IAI between 1st of January and 31st of October, 2016. All variables potentially associated with pre-defined outcomes: infection by a pathogen sensitive to non-pseudomonal cephalosporin or ciprofloxacin plus metronidazole (ATB 1, primary outcome), sensitive to piperacillin-tazobactam (ATB 2) and hospital mortality (secondary outcomes) were studied through logistic regression. Accuracy of the models was assessed by area under receiver operating characteristics (AUROC) curve and calibration was tested using the Hosmer-Lemeshow goodness-of-fit test.

**Results:**

Of 1804 patients screened 154 met inclusion criteria. Sensitivity to ATB 1 was independently associated with male gender (adjusted OR = 2.612) and previous invasive procedures in the last year (adjusted OR = 0.424) (AUROC curve = 0,65). Sensitivity to ATB 2 was independently associated with liver disease (adjusted OR = 3.580) and post-operative infections (adjusted OR = 2.944) (AUROC curve = 0.604). Hospital mortality was independently associated with age ≥ 70 (adjusted OR = 4.677), solid tumour (adjusted OR = 3.127) and sensitivity to non-pseudomonal cephalosporin or ciprofloxacin plus metronidazole (adjusted OR = 0.368).

The accuracy of pre-existing classifications to identify infection by a pathogen sensitive to ATB 1 was 0.59 considering place of acquisition, 0.61 infection extent and 0.57 local of infection, for ATB 2 it was 0.66, 0.50 and 0.57, respectively.

**Conclusion:**

None of existing classifications had a good discriminating power to identify IAIs caused by pathogens sensitive to current antibiotic treatment recommendations. A new classification, including patients’ individual characteristics like those included in the current model, might have a higher potential to distinguish IAIs by resistant pathogens allowing a better choice of empiric antibiotic therapy.

## Background

Intra-abdominal infections (IAIs) represent one of the most frequent gastrointestinal emergencies and a serious cause of morbidity and mortality [1–6]. A full classification that includes all facets of IAIs does not exist. An optimal classification designed for clinicians’ guidance in treatment should include: the origin of the source of infection, the anatomical extension, the supposed pathogens involved and risk factors for major resistance patterns, and the clinical condition of the patient [2, 6, 7].

IAIs are most frequently subdivided as uncomplicated or complicated, considering the extent of infection [1, 2, 6–11]. An uncomplicated intra-abdominal infection (uIAI) is an infection that involves a single organ and does not extend to the peritoneum [1, 2, 4–13]. A complicated intra-abdominal infection (cIAI) extends beyond a single organ into the peritoneal space [1, 2, 4–13].

Based on place of acquisition, infections are divided in: community-acquired intra-abdominal infection (CA-IAI) as an infection present at hospital admission or within 48 h in patients that did not meet the criteria for healthcare-associated infection and it is usually caused by the patient’s own flora [3, 6, 9, 12–15], hospital-acquired intra-abdominal infections (HA-IAIs), defined as infections that were not present at the time of hospital admission but emerge as noticeable after at least 48 h in patients hospitalized for other purpose than IAIs [12, 15–17], and healthcare-associated infections (HCA-IAIs) [18, 19], defined as infections present at hospital admission or within 48 h of admission in patients with previous contact with healthcare, namely invasive procedures, or that resides in a long-term care facility [12, 18].

The adequacy of initial empirical antibiotic therapy prescribed for IAIs is an essential need [1, 6, 9, 11, 12, 15], due to the minimum 48 h’ time required to have microbiological data available [1, 6, 9, 11]. Suitable empiric antibiotic therapy has a huge impact on the outcome of patients diagnosed with IAIs [12, 15, 20–22]. In CA-IAI, antibiotics with a restricted spectrum of activity are suggested [6, 9, 12, 23], such as cephalosporins without *Pseudomonas aeruginosa* coverage plus metronidazole, ciprofloxacin plus metronidazole, and piperacillin/tazobactam [6, 9, 12, 24]. In HA-IAIs, antibiotic regimens with vaster spectra of coverage are suggested [6, 9, 11].

There is evidence that inadequate and/or delayed antibiotic therapy is associated with treatment failure and increased mortality [1, 12, 13, 20–22, 25–30]. The sensitive equilibrium between the enhancement of empirical antibiotic therapy, which was proved to promote better clinical results, and the decline of needless antimicrobial overuse, which has been linked to the increasing development of multi-drug resistant (MDR) pathogens, is invariably needed when treating infections [6, 12, 31].

This study was designed to assess the impact of the different classifications of IAIs in the selection of the best antibiotic therapy, considering the need of adequate empirical antibiotic therapy and public health need to preserve antibiotics.

## Methodology

### Study design and selection

A retrospective cohort study developed at Hospital de Santo António, Centro Hospitalar Universitário do Porto, a tertiary care university hospital, including all adult patients (≥18 years old) discharged from the hospital with the diagnosis of IAI between 1st of January and 31st of October of 2016.

The main inclusion criteria were meeting the CDC definition of intra-abdominal infection (CDC. CDC/NHSN Surveillance Definitions for Specific Types of Infections. 2018; Available from: https://www.cdc.gov/nhsn/pdfs/pscmanual/17pscnosinfdef_current.pdf). First selection of the patients was achieved using the International Statistical Classification of Diseases and Related Health Problems 9th revision (ICD-9) including those with a discharge code compatible with intra-abdominal infection [ICD-9 codes considered in Additional file 1]. For selected patients, clinical file was reviewed to inclusion criteria confirmed and data of interest retrieved [see Additional file 2].

Exclusion criteria were: patients with anal/rectal pathology, infections caused by non-bacterial pathogens (that is virus, fungus and protozoa), non-complicated appendicitis, tuberculosis and patients with negative or no culture results.

The following variables were collected: age, gender, functional status, previous comorbidities, hospital admission date, discharge date, place of acquisition of the infection (community-acquired, healthcare-associated or hospital-acquired), extent of infection (uncomplicated or complicated), localization of infection (appendix, biliary tract, colon, small intestine, stomach/duodenum or other), post-operative infection (perforation, suture dehiscence, tertiary peritonitis, undetermined or non-applicable), microorganism(s) isolated, location of the isolated pathogen, realization of blood cultures, blood cultures’ result, resistance of isolated pathogens, empirical therapy administered to the patient in the first 24 h after diagnosis (antibiotic, daily dose, route of administration), change of the initial antibiotic therapy, reason of the changing, adequacy of the empirical antibiotic therapy, risk factors for healthcare-associated infections, previous colonization/infection by DR pathogen, previous antibiotic therapy (last 3 months), previous hospital admission (last year), previous invasive procedures (last year) and residence in a long-term care facility or nursing home and outcome at hospital discharge (dead or alive).

Functional status was assessed by the Karnofsky Performance Status Scale (KPS) [32] and previous comorbidities by the Charlson Comorbidity Index (CCI) [33]. The initial empirical antibiotic therapy was considered appropriate if all of the bacteria isolated from cultures were sensitive to at least one of the drugs administered.

The primary outcome of interest was infection by a pathogen sensitive to the following antibiotic scheme: non-pseudomonal cephalosporin or ciprofloxacin plus metronidazole. Secondary outcomes were sensitivity to piperacillin/tazobactam and hospital mortality.

### Statistical analysis

Data were described by means and standard deviations (SD) for continuous variables or with medians and inter-quartile ranges (IQR) if they showed a skewed distribution. Categorical variables were described with absolute frequencies and percentages. T-tests or Mann-Whitney-U tests were used to compare continuous variables. For categorical variables, these comparisons were performed using Pearson χ^2^ and Fisher’s exact tests.

All variables potentially associated with pre-defined outcomes were studied through logistic regression. Those with a clear association in the univariate analysis (*p* < 0.1) were included in the multivariable analysis. The results of the multivariable models are expressed as odds ratio (OR) with 95% confidence interval (CI_95%_) and *p*-values. The accuracy of the models was assessed by the area under receiver operating characteristics (AUROC) curve and calibration was tested using the Hosmer-Lemeshow goodness-of-fit test. The significance level was defined as *p* < 0.05.

Data were analysed using the Statistical Package for the Social Sciences (SPSS) version 25 for Windows (SPSS Inc., Chicago, IL, USA).

## Results

**Fig. 1:**
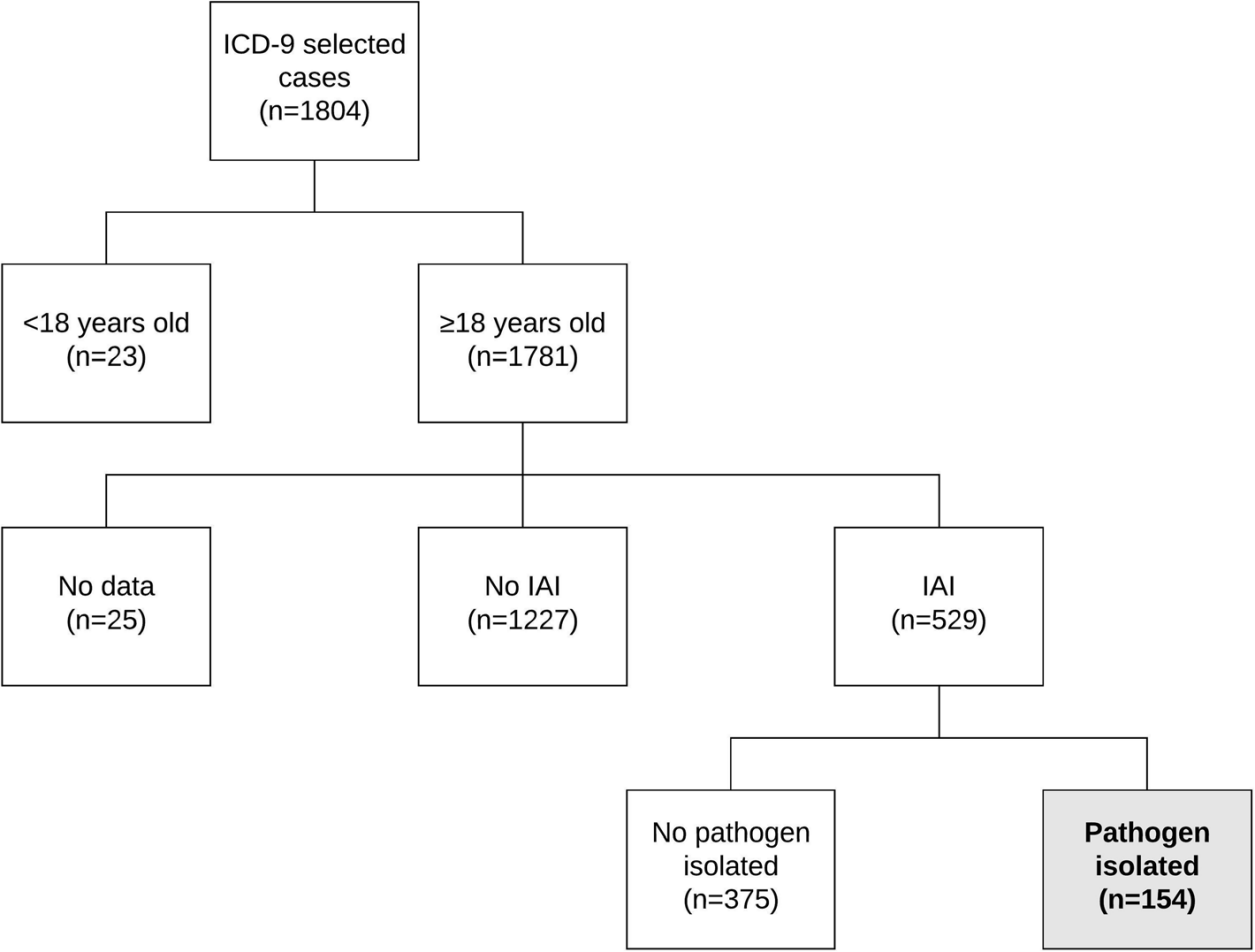
Flow diagram of the population selection process. (ICD-9, International Statistical Classification of Diseases and Related Health Problems 9th revision; IAI, intra-abdominal infection)

There were 1804 patients discharged from the hospital during the study period that met the ICD-9 established criteria. Of these, 154 met the inclusion criteria (Fig. 1).

Patients included in the study had a mean ± SD age of 73 ± 14 years and 48% were male; 112 (73%) need some help for daily activities defined by a KPS scale < 70 and 31 (20%) patients had no comorbidities according to the CCI definitions (Table 1). Median (IQR) hospital length of stay was 17 [10–29] days and hospital mortality rate was 22% (*n* = 34).

**Table 1:** Population’s general characteristics and risk factors for IAI by pathogens sensitive to ATB 1 or to ATB 2

	Total	IAI by pathogens susceptible to ATB 1	*p* value	IAI by pathogens susceptible to ATB 2	*p* value
Age ≥ 70, n (%)	101 (66)	52 (65)	0.874	72 (64)	0.253
Male gender, n (%)	74 (48)	46 (58)	0.015	49 (47)	0.614
Karnofsky Performance Status Scale < 70, n (%)	112 (73)	62 (78)	0.167	73 (70)	0.191
Diabetes, n (%)	45 (29)	19 (24)	0.121	28 (27)	0.308
Liver disease, n (%)	14 (9)	6 (8)	0.475	6 (6)	0.033
Solid tumour, n (%)	45 (29)	23 (29)	0.894	31 (30)	0.904
AIDS, n (%)	1 (1)	1 (1)	1.000^a^	1 (1)	1.000^a^
Chronic kidney disease, n (%)	26 (17)	9 (11)	0.052	18 (17)	0.900
Congestive heart failure, n (%)	11 (7)	7 (9)	0.537^a^	8 (8)	1.000^a^
Myocardial infarction, n (%)	10 (7)	7 (9)	0.331^a^	9 (9)	0.171^a^
Chronic obstructive pulmonary disease, n (%)	10 (7)	6 (8)	0.748^a^	8 (8)	0.504^a^
Peripheral vascular disease, n (%)	22 (14)	11 (14)	0.843	14 (13)	0.621
Cerebrovascular accident/transient ischemic attack, n (%)	14 (9)	8 (10)	0.683	11 (11)	0.550^a^
Dementia, n (%)	11 (7)	3 (4)	0.120^a^	7 (7)	1.000^a^
Hemiplegia, n (%)	1 (1)	1 (1)	1.000^a^	1 (1)	1.000^a^
Connective tissue disease, n (%)	2 (1)	1 (1)	1.000^a^	1 (1)	1.000^a^
Leukemia, n (%)	3 (2)	1 (1)	0.608^a^	2 (2)	1.000^a^
Malignant lymphoma, n (%)	1 (1)	1 (1)	1.000^a^	1 (1)	1.000^a^
Peptic ulcer disease, n (%)	4 (3)	3 (4)	0.621^a^	3 (3)	1.000^a^
Total score - Charlson Comorbidity Index, mean ± SD	5 ± 3	5 ± 3	0.744	5 ± 3	0.352
Residence in a long-term care facility or nursing home, n (%)	7 (5)	2 (3)	0.259^a^	4 (4)	0.681^a^
Previous colonization/infection by DR pathogen, n (%)	29 (19)	16 (20)	0.700	20 (19)	0.920
Previous antibiotic therapy, n (%)	39 (25)	14 (18)	0.020	22 (21)	0.068
Previous hospitalization, n (%)	82 (53)	37 (46)	0.070	51 (49)	0.089
Previous invasive procedures, n (%)	78 (51)	34 (43)	0.028	48 (46)	0.082
Post-operative Infection, n (%)	19 (12)	9 (11)	0.670	9 (9)	0.037

^a^Fisher’s exact test; AIDS, acquired immune deficiency syndrome; ATB 1, non-pseudomonal cephalosporin or ciprofloxacin plus metronidazole; ATB 2, piperacillin/tazobactam; *DR* drug-resistant, *OR* odds ratio, *SD* standard deviation

The following risk factors were associated with an IAI by pathogens sensitive to ATB 1: male gender, previous antibiotic therapy and previous invasive procedures (Table 1). The final model retained male gender with an adjusted OR (CI_95%_) = 2.612 (1.328-5.148) and previous invasive procedures in the last year with an adjusted OR (CI_95%_) = 0.424 (0.216-0.833). The AUROC curve (CI_95%_) was 0.65 (0.57-0.74) (Table 2).

Considering ATB 2 as the dependent variable, the final model retained liver disease with an adjusted OR (CI_95%_) = 3.580 (1.126–10.879) and post-operative infections with an adjusted OR (CI_95%_) = 2.944 (1.096–7.908) (Table 1). The AUROC curve (CI_95%_) was 0.604 (0.504–0.704) (Table 2).

### Intra-abdominal infections classification and microbiological results

Comparison of the accuracy of different classifications of the IAI in determining infection by a pathogen sensitive to current treatment guidelines is shown in Table 2.

**Table 2:** Discriminative power of each classification for IAI by pathogen sensitive to antibiotic scheme for CA-IAI

Classification of intra-abdominal infection	AUROC curve (95% CI) for ATB 1	AUROC curve (95% CI) for ATB 2
Place of acquisition: CA-IAI, HCA-IAI, HA-IAI	0.59 (0.50–0.68)	0.66 (0.56–0.75)
Place of acquisition: CA-IAI and HA-IAI	0.54 (0.45–0.63)	0.59 (0.49–0.69)
Extent of infection: uIAI or cIAI	0.61 (0.52–0.70)	0.50 (0.40–0.60)
Local of infection	0.57 (0.48–0.66)	0.57 (0.48–0.67)
Current model	0.65 (0.57–0.74)	0.60 (0.50–0.70)

*AUROC* area under receiver operating characteristics, *CI* confidence interval, *ATB I*, non-pseudomonal cephalosporin or ciprofloxacin plus metronidazole, *ATB 2* piperacillin/tazobactam, *CA-IAI* community-acquired intra-abdominal infection, *HCA-IAI* healthcare-associated intra-abdominal infection, *HA-IAI* hospital-acquired intra-abdominal infection, *uIAI* uncomplicated intra-abdominal infection, *cIAI* complicated intra-abdominal infection

Postoperative infection was observed in 19 (12%) patients, caused by suture dehiscence in 8 (5%) patients, perforation in 3 (2%) patients and undetermined or by other causes in 8 (5%) patients.

Blood cultures were drawn in 122 (79%) patients at hospital admission, of those 69 (57%) were positive. Besides blood, pathogens were isolated from peritoneal fluid in 60 (39%), bile in 13 (8%), faeces in 11 (7%), abscess in 9 (5%), biliary drainage fluid in 1 (1%) and pancreas drainage fluid in 1 (1%).

The microbiological profile according to different classifications is shown in Table 3.

### Antibiotic therapy

Although non-pseudomonal cephalosporin or ciprofloxacin plus metronidazole is the shorter spectrum recommended antibiotic therapy for CA-IAI, it was administered to only one patient, but if it was administered to all patients, it would have been adequate in 54% of them.

Among the antibiotic agents administered, piperacillin/tazobactam was the most frequently used, in 123 (80%) patients, followed by metronidazole in 7 (5%), imipenem plus cilastatin in 6 (4%), ciprofloxacin in 5 (3%) and amoxicillin/clavulanic acid in 4 (3%). Nevertheless, the sensitivity to piperacillin/tazobactam was exhibited in 105 (68%) patients.

The distribution of sensitive pathogens to the studied antibiotic regimens (non-pseudomonal cephalosporin or ciprofloxacin plus metronidazole, or piperacillin/tazobactam), according to the different classifications is shown in Table 3.

**Table 3:** Microbiological profile of IAI according to the different classifications

Place of acquisition, n (%)	CA-IAI, 83 (54)	HCA-IAI, 45 (29)	HA-IAI, 26 (17)
Microbiological profile, n (%)	Monomicrobial, 46 (55) * E. coli*, 19 (41) *Klebsiella* spp., 7 (15) * E. faecium*, 4 (19) Other, 16 (25) Polymicrobial, 37 (45)	Monomicrobial, 32 (61) *E. coli*, 7 (22) *Klebsiella* spp., 7 (22) *Clostridium* spp., 6 (19) Other, 12 (37) Polymicrobial, 13 (29)	Monomicrobial, 17 (65) *E. coli*, 4 (24) *Klebsiella* spp., 2 (12) *E. faecium*, 2 (12) *Clostridium* spp., 2 (12) Other, 7 (40) Polymicrobial, 9 (35)
IAI by pathogens sensitive to ATB I, n (%)	50 (60)	20 (44)	10 (40)
IAI by pathogens sensitive to ATB 2, n (%)	66 (80)	27 (60)	12 (46)
Extent of infection, n (%)		uIAI, 25 (16)	cIAI, 129 (84)
Microbiological profile, n (%)		Monomicrobial, 15 (60) *Clostridium* spp., 9 (60) *E. coli*, 2 (13) *E. faecium*, 2 (13) Other, 2 (13) Polymicrobial, 10 (40)	Monomicrobial, 80 (62) *E. coli*, 28 (35) *Klebsiella* spp., 16 (20) *E. faecium*, 8 (10) Other, 28 (35) Polymicrobial, 49 (38)
IAI by pathogens sensitive to ATB I, n (%)		5 (20)	75 (60)
IAI by pathogens sensitive to ATB 2, n (%)		17 (68)	88 (68)
Local of infection, n (%)	Biliary Tract, 78 (51)	Colon, 43 (28)	Other, 33 (21)
Microbiological profile, n (%)	Monomicrobial, 54 (69) *E. coli*, 23 (43) *Klebsiella* spp., 13 (24) *E. faecium*, 7 (13) Other, 35 (45) Polymicrobial, 24 (31)	Monomicrobial, 21 (49) *Clostridium* spp., 11 (52) *E. coli*, 3 (14) *E. faecium*, 2 (10) Other, 5 (24) Polymicrobial, 22 (51)	Monomicrobial, 20 (60) Polymicrobial, 13 (40)
IAI by pathogens sensitive to ATB I, n (%)	42 (54)	20 (47)	18 (55)
IAI by pathogens sensitive to ATB 2, n (%)	51 (65)	32 (74)	22 (67)

ATB I, non-pseudomonal cephalosporin or ciprofloxacin plus metronidazole; ATB 2, piperacillin/tazobactam; CA-IAI, community-acquired intra-abdominal infection; HCA-IAI, healthcare-associated intra-abdominal infection; HA-IAI, hospital-acquired intra-abdominal infection; uIAI, uncomplicated intra-abdominal infection; cIAI, complicated intra-abdominal infection

The empirical antibiotic therapy was changed in 83 (54%) patients, of these adjustments to the susceptibility profile of the isolated pathogen was the most frequent reason in 40 (48%) patients.

The initial antibiotic therapy was adequate in 98 (64%) patients: 60 (72%) with CA-IAIs, 26 (56%) with HCA-IAIs and 12 (46%) with HA-IAIs (*p* = 0.034). In uIAI, 17 (68%) patients had adequate initial antibiotic therapy and in cIAI 81 (63%) patients (*p* = 0.620). There was no relation between the local of infection and the adequacy of the initial antibiotic therapy (*p* = 0.628).

In patients with post-operative infections, a higher frequency of inadequate antibiotic therapy was observed (63% vs. 33%, *p* = 0.010).

### Prognostic risk factors in intra-abdominal infections

Factors significantly associated with hospital mortality were: KPS score < 70, chronic kidney disease, the total score of CCI, localization of infection, polymicrobial flora and sensitivity to non-pseudomonal cephalosporin or ciprofloxacin plus metronidazole (Table 4).

**Table 4:** Risk factors for hospital mortality

	Total	Hospital mortality	*p* value	Crude OR
Age ≥ 70, n (%)	101 (66)	27 (27)	0.055	2.398
Male gender, n (%)	74 (48)	13 (18)	0.194	0.599
Karnofsky Performance Status Scale < 70, n (%)	112 (73)	20 (18)	0.042	0.435
Diabetes, n (%)	45 (29)	10 (22)	0.978	0.988
Liver disease, n (%)	14 (9)	4 (29)	0.512^a^	1.467
Solid tumour, n (%)	45 (29)	14 (31)	0.082	2.010
AIDS, n (%)	1 (1)	0 (0)	1.000^a^	–
Chronic kidney disease, n (%)	26 (17)	10 (39)	0.027	2.708
Congestive heart failure, n (%)	11 (7)	0 (0)	0.124^a^	–
Myocardial infarction, n (%)	10 (7)	1 (10)	0.461^a^	0.374
Chronic obstructive pulmonary disease, n (%)	10 (7)	3 (30)	0.693^a^	1.562
Peripheral vascular disease, n (%)	22 (14)	4 (18)	0.785^a^	0.756
Cerebrovascular accident/transient ischemic attack, n (%)	14 (9)	0 (0)	0.041^a^	–
Dementia, n (%)	11 (7)	5 (46)	0.066^a^	3.276
Hemiplegia, n (%)	1 (1)	0 (0)	1.000^a^	–
Connective tissue disease, n (%)	2 (1)	1 (50)	0.394^a^	3.606
Leukemia, n (%)	3 (2)	2 (67)	0.123^a^	7.437
Malignant lymphoma, n (%)	1 (0,6)	0 (0)	1.000^a^	–
Peptic ulcer disease, n (%)	4 (3)	0 (0)	0.576^a^	–
Total Score - Charlson Comorbidity Index, mean ± SD	5 ± 3	5 ± 3	0.045	0.866 per point
Residence in a long-term care facility or nursing home, n (%)	7 (5)	2 (29)	0.469^a^	1.484
Previous colonization/infection by DR pathogen, n (%)	29 (19)	4 (14)	0.322^a^	0.507
Previous antibiotic therapy, n (%)	39 (25)	11 (28)	0.286	1.571
Previous hospitalization, n (%)	82 (53)	19 (23)	0.727	1.146
Previous invasive procedures, n (%)	78 (51)	18 (23)	0.644	1.200
Initial antibiotic therapy adequate, n (%)	98 (64)	21 (21)	0.797	0.902
Polymicrobial flora, n (%)	59 (38)	18 (31)	0.049	2.168
Sensitive to ATB I, n (%)	80 (52)	12 (15)	0.030	0.417
Sensitive to ATB 2, n (%)	105 (68)	19 (18)	0.084	0.501
Positive blood cultures, n (%)	69 (57)	16 (15)	0.065	0.429
Post-operative infection, n (%)	19 (12)	3 (16)	0.570^a^	1.590
Place of acquisition – classification, n (%)	154 (100)	34 (22)	0.790	–
Community-acquired, n (%)	83 (54)	18 (22)	–	1.000
Healthcare-associated, n (%)	45 (29)	9 (20)	–	1.330
Hospital-acquired, n (%)	26 (17)	7 (27)	–	1.474
Extent of infection – classification, n (%)	154 (100)	34 (22)	0.195^a^	–
Uncomplicated, n (%)	25 (16)	3 (12)	–	1.000
Complicated, n (%)	129 (84)	31 (24)	–	2.320

^a^Fisher’s exact test; *AIDS* acquired immune deficiency syndrome, *DR* drug-resistant, *OR* odds ratio, *ATB I* non-pseudomonal cephalosporin or ciprofloxacin plus metronidazole, *ATB 2* piperacillin/tazobactam

In the multivariable analysis with the hospital mortality as the dependent variable, the final model retained age ≥ 70 with an adjusted OR (CI_95%_) = 4.677 (1.260–17.358), solid tumour with an adjusted OR (CI_95%_) = 3.127 (1.183–8.266) and sensitivity to non-pseudomonal cephalosporin or ciprofloxacin plus metronidazole with an adjusted OR (CI_95%_) = 0.368 (0.138–0.980).

## Discussion

The main finding of our study was the fact that none of the existing classifications had a good discriminating power to identify IAIs caused by pathogens sensitive to the current antibiotic treatment recommendations. This supports the poor utility of the existing classifications of IAI.

Independent risk factors for IAI caused by those pathogens was male gender, for which we cannot find an explanation or similar results in other published studies; and previous invasive procedures in the last year, which were associated with IAI caused by pathogens not sensitive to the shorter antibiotic scheme recommended for CA-IAI, which has also been enlightened by other studies that linked previous invasive procedures with greater rates of colonization and infection with MDR pathogens [18].

Caution should be taken when using antibiotic schemes containing fluroquinolones due to its high potential to induce resistance and its very high resistance rates.

An alternative antibiotic scheme for CA-IAI is piperacillin/tazobactam, which is an antibiotic with a broader spectrum. Postoperative infections were associated with a higher sensitivity to this antibiotic therapy. This result cannot be explained by this study or the literature reviewed. Liver disease also showed an association with increased sensitivity to this antibiotic therapy. Sargenti et al. [34] revealed that patients with liver disease have mainly HCA-IAIs and HA-IAIS, which are often caused by bacteria resistant to commonly used antibiotics, piperacillin/tazobactam might be an option for this group of patients [35].

Our data revealed that age ≥ 70 years was associated to an increased hospital mortality, which is also supported by the conclusions of other studies [15]. Higher age was also connected, by many studies, to a greater prevalence of DR pathogens, which has been implicated in an augmented mortality rate [36–38]. Solid tumour was the comorbidity that presented a connection with greater mortality, which can be clarified by the higher prevalence of DR pathogens that will result in an increased mortality rate [12, 36–38]. Patients with pathogens sensitive to non-pseudomonal cephalosporin or ciprofloxacin plus metronidazole had an associated lower hospital mortality, in this study. This antibiotic scheme recommended for community-acquired infections is the shortest spectrum one, pathogens sensitive to this regimen are less resistant, having adequate antibiotic therapy more frequently and causing infection with lower severity, which can explain this association.

The total score of CCI was associated with increased hospital mortality in accordance with the study by Montravers et al. [15], that also revealed that the presence of one or more comorbidities had a predictive value for hospital mortality.

In our study, most of the patients had blood cultures taken (79%) and more than half were positive (57%), which is similar to the results of Krobot et al. [21] (43%), but considerably higher than the data observed by Montravers et al. [15] (6%). Microbiological cultures, including blood cultures, in IAIs are extremely important to establish an adequate antibiotic therapy and should be collected in every patient with IAI [3, 9, 12].

The distribution of isolated pathogens, in our study, was identical to other reports, being *Escherichia coli* the most frequent, independently of the classification used [5, 15, 21, 39–41]. The prevalence of monomicrobial IAIs (62%) was much superior in comparison to the studies by Claridge et al. [41] (41%) and Shah et al. [42] (33%).

In our study the rate of inadequate antibiotic therapy (36%) was within the range described in similar studies: between 13 and 44% [21]. and it was not associated with increased hospital mortality which was also observed by Montravers et al. [15], but opposed to other several studies [5, 13, 30]. Timing and adequacy of source control are recognized as major prognostic factors and those were not targeted in our study and might explain this finding.

The hospital mortality observed in this study is higher than the described by Sartelli et al. [40] (22% vs. 11%), but comparable to the results presented by Montravers et al. [15]: 22% vs. 24% in CA-IAIs; 20% in HCA-IAIs and 27% vs. 23% in nosocomial IAIs.

Our study has some limitations. Firstly, since it is a single-centred study, the analysis was based on local data and resistance patterns. Being a retrospective study, data collection was limited to the existing records. Although all variables thought to be relevant were collected in the present study, there is always the possibility of additional variables not considered here to be linked to the outcome studied. Therefore, the results of this study must be interpreted prudently.

We have not found studies that investigated the discrimination of different classifications of IAI on the selection of the best antibiotic therapy, so we assume that our results could be of value to the clinicians in the field.

## Conclusions

In our study, none of the classifications revealed good accuracy in determining infection by a pathogen sensitive to current treatment guidelines, although gender, comorbidities (namely chronic liver disease, previous invasive procedures and post-operative infections) were significantly associated with sensitivity to those antibiotic schemes.

A new classification of IAI that includes patient’s risk factors might have a greater potential in distinguish IAIs by sensitive pathogens allowing a better choice of empiric antibiotic therapy, tailored to individual patient needs with the minimum selective pressure.

## Supplementary information

**Additional file 1.** ICD-9 selected codes. Selected codes of the international statistical classification of diseases and related health problems 9th revision (ICD-9) to achieve the first selection of patients.

**Additional file 2.** Case report form. The case report form created and used to collect the data from the final selection of patients.

## Data Availability

The datasets generated and analysed during the current study are not publicly available since they are currently in use for other studies, but are available from the corresponding author on reasonable request.

## Abbreviations

AIDS: Acquired immune deficiency syndrome
ATB 1: Non-pseudomonal cephalosporin or ciprofloxacin plus metronidazole
ATB 2: Piperacillin/tazobactam
ATB: Antibiotic
AUROC: Area under receiver operating characteristics
CA-IAI: Community-acquired intra-abdominal infection
CCI: Charlson Comorbidity Index
CHUP: Centro Hospitalar Universitário do Porto
CI_95%_: 95% Confidence interval
cIAI: Complicated intra-abdominal Infection
DR: Drug-resistant
HA-IAI: Hospital-acquired intra-abdominal infection
HCA-IAI: Healthcare-associated intra-abdominal infection
IAI: Intra-abdominal infection
ICD-9: International Statistical Classification of Diseases and Related Health Problems 9th revision
IQR: Inter-quartile range
KPS: Karnofsky Performance Status Scale
MDR: Multidrug-resistant
OR: Odds ratio
ROC: Receiver operating characteristics
SD: Standard deviation
SPSS: Statistical Package for the Social Sciences
uIAI: Uncomplicated intra-abdominal infection
