# Supplementary material for: Intra-abdominal infections: the role of different classifications on the selection of the best antibiotic treatment
Source: BMC Infect Dis. 2019 Nov 21;19:980. doi: 10.1186/s12879-019-4604-0 (PMC6873447; doi:10.1186/s12879-019-4604-0)
Supplement: Supplementary file 1 — Additional file 1. ICD-9 selected codes. Selected codes of the international statistical classification of diseases and related health problems 9th revision (ICD-9) to achieve the first selection of patients. [file 12879_2019_4604_MOESM1_ESM.pdf]

ICD-9 selected codes

ICD-9 code	Designation
001	Cholera disease
002	Typhoid and paratyphoid fevers
003	Other Salmonella infections
004	Shigellosis
005	Other poisoning (bacterial)
008	Intestinal infections due to other organisms
009	Ill-defined intestinal infections
038.3	Septicemia due to anaerobes
038.4	Septicemia due to other gram-negative organisms
038.8	Other specified septicemias
038.9	Unspecified septicemia
540	Acute appendicitis
562.1	Diverticula of colon
567	Peritonitis and retroperitoneal infections
568.89	Other specified disorders of peritoneum
569.5	Abscess of intestine
569.61	Infection of colostomy or enterostomy
569.83	Perforation of intestine
572.0	Acute and subacute necrosis of liver
574.0	Calculus of gallbladder with acute cholecystitis
574.1	Calculus of gallbladder with other cholecystitis
574.3	Calculus of bile duct with acute cholecystitis
574.4	Calculus of bile duct with other cholecystitis
574.6	Calculus of gallbladder and bile duct with acute cholecystitis
574.7	Calculus of gallbladder and bile duct with other cholecystitis
574.8	Calculus of gallbladder and bile duct with acute and chronic cholecystitis
575.0	Acute cholecystitis
575.1	Other cholecystitis
576.1	Cholangitis
576.3	Perforation of bile duct
577	Diseases of pancreas
