# Supplementary material for: Intra-abdominal infections: the role of different classifications on the selection of the best antibiotic treatment
Source: BMC Infect Dis. 2019 Nov 21;19:980. doi: 10.1186/s12879-019-4604-0 (PMC6873447; doi:10.1186/s12879-019-4604-0)
Supplement: Supplementary file 2 — Additional file 2. Case report form. The case report form created and used to collect the data from the final selection of patients. [file 12879_2019_4604_MOESM2_ESM.pdf]

Intra-abdominal Infections (IAIs): the role of different classifications on the selection of the best antibiotic treatment

Case Report Form

Date *(DD/MM/YYYY)*	

Assigned Study Number	

Assigned Study Number - **Completion Instructions**:	
1st space	Initial of Patient's First Name
2nd space	Initial of Patient's Last Name
3rd space	Initial of Hospital Department's Name
4th space	2nd letter of Hospital Department's Name
5th space	Hospital Department Number, when applicable
6th-9th spaces	Case Number counting

1. General information

1. Gender	1		Male	2		Female

2. Hospital admission date *(DD/MM/YYYY)*	
3. Discharge date *(DD/MM/YYYY)*	

2. General Medical Condition

1. Karnofsky Performance Status Scale			
1	**100**	Normal with no complaints; no evidence of disease.	
2	**90**	Able to carry on normal activity; minor signs or symptoms of disease.	
3	**80**	Normal activity with effort; some signs or symptoms of disease.	
4	**70**	Cares for self; unable to carry on normal activity or to do active work.	
5	**60**	Requires occasional assistance, but able to care for most of his personal needs.	
6	**50**	Requires considerable assistance and frequent medical care.	
7	**40**	Disabled; requires special care and assistance.	
8	**30**	Severely disabled; hospital admission is indicated although death not imminent.	
9	**20**	Very sick; hospital admission necessary; active supportive treatment necessary.	

1. Karnofsky Performance Status Scale			
10	**10**	Moribund; fatal processes progressing rapidly.	
11	**0**	Dead	

3. Charlson Comorbidity Index (CCI)

1. Age				Points
1	< 50 years		0	
2	50-59 years		+1	
3	60-69 years		+2	
4	70-79 years		+3	
5	> 80 years		+4	

2. Diabetes mellitus				Points
1	None		0	
2	Uncomplicated		+1	
3	End-organ damage		+2	

3. Liver disease				Points
1	None		0	
2	Mild		+1	
3	Moderate to severe		+3	

4. Solid tumor				Points
1	None		0	
2	Localized		+2	
3	Metastatic		+6	

5. AIDS				Points
1	No		0	
2	Yes		+6	

6. Moderate to severe Chronic Kidney Disease				Points
1	No	☐	0	
2	Yes	☐	+2	

7. Congestive Heart Failure				Points
1	No	☐	0	
2	Yes	☐	+1	

8. Myocardial infarction				Points
1	No	☐	0	
2	Yes	☐	+1	

9. Chronic Obstructive Pulmonary Disease				Points
1	No	☐	0	
2	Yes	☐	+1	

10. Peripheral vascular disease				Points
1	No	☐	0	
2	Yes	☐	+1	

11. Cerebrovascular Accident/Transient Ischemic Attack				Points
1	No	☐	0	
2	Yes	☐	+1	

12. Dementia				Points
1	No	☐	0	
2	Yes	☐	+1	

13. Hemiplegia				Points
1	No	☐	0	

2	Yes		+2	

14. Connective tissue disease				Points
1	No		0	
2	Yes		+1	

15. Leukemia				Points
1	No		0	
2	Yes		+2	

16. Malignant lymphoma				Points
1	No		0	
2	Yes		+2	

17. Peptic ulcer disease				Points
1	No		0	
2	Yes		+1	

18. **TOTAL SCORE**	

4. Infection's classification

1. Place of acquisition		
**Community-acquired:** infections that were present at hospital admission or within 48 hours of admission that did not met criteria for healthcare-associated infection.	1	
**Healthcare-associated:** infections that were present at hospital admission or within 48 hours of admission that met one of the following criteria:<br>- intravenous therapy took at home, wound treatment or specialized nursing care through an healthcare agency, family or friends;<br>- had self-delivered intravenous medical therapy within 30 days previous to the infection;<br>- attended an hospital or haemodialysis clinic or intravenous chemotherapy received 30 days before;<br>- had an hospitalization in an acute care hospital for at least 2 days in the past 90 days;<br>- inhabited in a nursing home or long-term care facility.	2	

1. Place of acquisition		
**Hospital-acquired:** infections that were not present or incubating at the time of hospital admission but that become evident after the first 48 hours.	3	

2. Extent of Infection		
**Uncomplicated IAI:** an infection that only involves a single organ and does not extend to the peritoneum.	1	
**Complicated IAI:** an infection that extends beyond a single organ into the peritoneal space, causing peritoneal inflammation.	2	

3. Localization of infection

Appendix	1	
Biliary Tract	2	
Colon	3	
Small Intestine	4	
Stomach/Duodenum	5	
Other:____	6	

4. Post-operative Infection

Perforation	1	
Suture Dehiscence	2	
Tertiary Peritonitis	3	
Undetermined	4	
Other:____	5	
Non Applicable	9999	

5. Microorganisms

Aerobes		
Gram-negative bacilli		
Enterobacter spp.	1	
Escherichia coli	2	

Aerobes		
Gram-negative bacilli		
Klebsiella spp.	3	
Proteus mirabilis	4	
Pseudomonas aeroginosa	5	
Salmonella spp.	6	
Other:____	7	
Non Applicable	9999	

Gram-positive cocci		
Coagulase-negative *Staphylococcus*	8	
Enterococcus faecalis	9	
Enterococcus faecium	10	
Enterococcus (other)	11	
Staphylococcus aureus	12	
Streptococcus spp.	13	
Other:____	14	
Non Applicable	9999	

Anaerobes		
Peptostreptococcus spp.	15	
Bacteroides spp.	16	
Clostridium spp.	17	
Other:____	18	
Non Applicable	9999	

19. **Where** was the **pathogen isolated**? ____

20. **Were** blood cultures **taken**?	1		Yes	2		No
21. **Positive** blood cultures?	1		Yes	2		No

6. Resistance of isolated pathogens

**Pathogen**	Penicilina G	Ampicilina	Met/Oxacilina	Piperacilina/Tazob	Amox/Ac Clav	Cefalosporinas 1ªG	Cefoxitina	Cefuroxime	Ceftazidima	Cefotaxima	Cefepime	Tigeciclina	Imip/Meropnem	Ertapenem	Aztreonam	Eritromicina	Azitromicina	Clindamicina	Metronidazol	Vancomicina	Teicoplanina	Amicacina	Gentamicina	Tobramicina	Estreptomicina AC	Ciprofloxacina	Levofloxacina	Cotrimoxazol	Tetraciclina	Linezolide	Rifampicina	Colistin
1.																																
2.																																
3.																																
4.																																

Legend - **Microorganisms**	
**1**	
**2**	
**3**	
**4**	

7. Treatment

1. Empirical therapy administered to the patient in the first 24 hours after diagnosis		
Antibiotic	Daily dose (total amount given in the first 24 hours)	Route of administration

2. Was the **initial** antibiotic therapy **changed**?	1 ☐	Yes	2 ☐	No

2.1. If **it was**, please **specify** the reason for change:	
Clinical deterioration	1 ☐
Directed towards sensitivity profile of isolated pathogen	2 ☐

2.1. If **it was**, please **specify** the reason for change:		
No evidence of infection	3	
Side effects	4	
Other: ____	5	
Non Applicable	9999	

3. Was the initial empiric antibiotic therapy "**adequate**"?	1		Yes	2		No

If the initial antibiotic prescribed within 24 hours matched in vitro susceptibility of a pathogen deemed to be likely cause of infection and when the dosage and route of administration are appropriate for current medical status (focus and severity of infection); only patients with positive microbiology are considered in this analysis

8. Risk factors for healthcare-associated infection

1. Previous colonization/infection by DR pathogen	1		Yes	2		No	3		Unknown
2. Previous antibiotic therapy (last 3 months)	1		Yes	2		No	3		Unknown
3. Previous hospital admission (last year)	1		Yes	2		No	3		Unknown
4. Previous invasive procedures (last year)	1		Yes	2		No	3		Unknown
5. Residence in a long-term care facility or nursing home	1		Yes	2		No	3		Unknown

9. Outcome

1. At hospital discharge	1		Dead	2		Alive
